# Suppression of metastasis-associated S100A4 gene expression by gamma-interferon in human colon adenocarcinoma cells

**DOI:** 10.1038/sj.bjc.6690331

**Published:** 1999-04

**Authors:** K Takenaga

**Affiliations:** Division of Chemotherapy, Chiba Cancer Center Research Institute, 666-2 Nitona, Chuoh-ku, Chiba 260-8717, Japan

**Keywords:** IFN-γ, S100A4, IFN-γ-receptor, motility, colon carcinoma

## Abstract

S100A4 belongs to the S100 subfamily of calcium-binding proteins and has been suggested to be directly involved in invasion and metastasis of rodent and human tumour cells. The present study demonstrates that interferon gamma (IFN-γ), but not IFN-α and IFN-β, down-regulates the S100A4 mRNA level in colon adenocarcinoma WiDr cells in time- and dose-dependent manners. The effect was not associated with any cytotoxicity and was specific for the S100A4 mRNA, since the levels of the S100A6 and GAPDH mRNAs were not significantly affected by the treatment. IFN-γ also strongly suppressed the S100A4 mRNA expression in HT-29 cells, but weakly in Colo201 cells. Flow cytometric analysis revealed that the level of the IFN-γ receptor expression in Colo201 cells was lower than that in WiDr and HT-29 cells, suggesting that the suppression of the S100A4 expression by IFN-γ depends on the amount of cell surface IFN-γ receptor protein. IFN-γ had no effect on the transcription rate of the S100A4 gene but reduced the stability of the S100A4 mRNA. WiDr cells treated with IFN-γ showed reduced motile ability, further supporting the assumption that the S100A4 gene product is involved in controlling cell motility. © 1999 Cancer Research Campaign


*British Journal of Cancer* (1999) **80**(1/2), 127–132
© 1999 Cancer Research Campaign
Article no. bjoc.1998.0331

### Suppression of metastasis-associated S100A4 gene expression by gamma-interferon in human colon adenocarcinoma cells

**K Takenaga**

Division of Chemotherapy, Chiba Cancer Center Research Institute, 666-2 Nitona, Chuoh-ku, Chiba 260-8717, Japan

**Summary** S100A4 belongs to the S100 subfamily of calcium-binding proteins and has been suggested to be directly involved in invasion and metastasis of rodent and human tumour cells. The present study demonstrates that interferon gamma (IFN-γ), but not IFN-α and IFN-β, down-regulates the S100A4 mRNA level in colon adenocarcinoma WiDr cells in time- and dose-dependent manners. The effect was not associated with any cytotoxicity and was specific for the S100A4 mRNA, since the levels of the S100A6 and GAPDH mRNAs were not significantly affected by the treatment. IFN-γ also strongly suppressed the S100A4 mRNA expression in HT-29 cells, but weakly in Colo201 cells. Flow cytometric analysis revealed that the level of the IFN-γ receptor expression in Colo201 cells was lower than that in WiDr and HT-29 cells, suggesting that the suppression of the S100A4 expression by IFN-γ depends on the amount of cell surface IFN-γ receptor protein. IFN-γ had no effect on the transcription rate of the S100A4 gene but reduced the stability of the S100A4 mRNA. WiDr cells treated with IFN-γ showed reduced motile ability, further supporting the assumption that the S100A4 gene product is involved in controlling cell motility.

**Keywords:** IFN-γ; S100A4; IFN-γ-receptor; motility; colon carcinoma

S100A4, also known as CAPL, pEL98, mts1, p9Ka, 18A2, 42A and FSP1, is a member of the S100 subfamily of calcium-binding proteins (Hilt and Kligman, 1991; Celio et al, 1996; Schäfer and Heinzmann, 1996). Its expression has been shown to be associated with cell immortalization (Goto et al, 1988), cell growth (Jackson-Grusby et al, 1987), differentiation of myoepithelial cells (Barraclough et al, 1987) and promyelocytic leukaemia cells (Takenaga et al, 1994*b*), fibrogenesis (Strutz et al, 1995) and tumour progression (Grigorian et al, 1996). In addition, several lines of evidence indicate that S100A4 is directly involved in tumour cell invasion and metastasis. Ebralitze et al reported that the expression of S100A4 was elevated in metastatic tumour cells (Ebralidze et al, 1989). We and others have shown that the increased expression of S100A4 in tumour cells by introduction of the S100A4 gene resulted in an enhancement of cell motility and invasive and metastatic potential of tumour cells (Davies et al, 1993; Parker et al, 1994; Takenaga et al, 1994*a*). On the other hand, reduction of the expression of S100A4 by means of anti-sense S100A4 RNA or anti-S100A4 ribozyme in high-metastatic tumour cells resulted in the suppression of their invasive and metastatic capabilities (Grigorian et al, 1993; MæLandsmo et al, 1996; Takenaga et al, 1997*a*). Although the precise functions of the S100A4 protein are not fully understood, recent studies implicate that the protein interacts with cytoskeletal proteins such as non-muscle tropomyosins (Takenaga et al, 1994*c*, 1994*d*) and myosin (Kriajevska et al, 1994; Ford and Zain, 1995) in a $Ca^{2+}$-dependent manner and thereby modulates cell motility (Takenaga et al, 1994*a*, 1994*d*; Ford and Zain, 1995). Importantly, several studies have shown that S100A4 may also play a role in invasion and metastasis of human tumours including breast (Pedrocchi et al, 1994; Ilg et al, 1996) and colorectal carcinomas (Ilg et al, 1996; Takenaga et al, 1997*b*).

Metastasis comprises a complex series of interactions between tumour cells and their environment (Radinsky, 1995). At each step, tumour cells are exposed to a variety of local factors such as hormones, growth factors and cytokines. These factors undoubtedly modulate the metastasis-associated properties of tumour cells such as growth potential, secretion of type IV collagenolytic activity, cell migration, cell adhesion and natural killer (NK) cell sensitivity. Since it is largely unknown whether the S100A4 expression in tumour cells is also influenced by such factors, the effects of various growth factors and cytokines on the expression of S100A4 in human colon adenocarcinoma cells were examined. In this study, the fact that interferon gamma (IFN-γ) efficiently suppresses the S100A4 expression and reduces the motile ability in the cells is demonstrated.

*Received 20 May 1998*
*Revised 11 November 1998*
*Accepted 14 November 1998*

*Correspondence to:* K Takenaga

### MATERIALS AND METHODS

### Reagents

Recombinant human IFN-α (specific activity; $3 \times 10^8$ IU $mg^{-1}$) and IFN-γ (specific activity; $3 \times 10^7$ IU $mg^{-1}$) were purchased from Pepro Tech EC Ltd (London, UK). Human IFN-β (specific activity; $1 \times 10^5$ IU $mg^{-1}$) and mouse monoclonal anti-human IFN-γ receptor (clone GIR-208) were obtained from Sigma Chemicals Co. (St Louis, MO, USA). Recombinant human tumour necrosis factor-α (TNF-α) was purchased from Gibco BRL (Grand Island, NY, USA).

### Cell culture

Human colon adenocarcinoma cell lines, WiDr, Colo201 and HT-29, were obtained from the American Type Culture Collection (Rockville, MD, USA). They were cultured in RPMI-1640 medium supplemented with 10% heat (56°C, 30 min)-inactivated fetal bovine serum, 100 μg $ml^{-1}$ streptomycin and 100 units per ml penicillin.

### Western blot analysis

Cells were lysed in extraction buffer (150 mM sodium chloride, 50 mM Tris-HCl (pH 7.5), 1% Triton X-100, 1 mM EGTA and 1 mM phenylmethylsulphonyl fluoride) (Takenaga et al, 1994*a*). Cell lysates were centrifuged at 10 000 ***g*** for 10 min, and the supernatant was used for immunoblot analysis. Immunoblot analysis was carried out as described previously (Takenaga et al, 1994*a*) using anti-S100A4 polyclonal antibody and an enhanced chemiluminescence Western blotting detection kit (Amersham).

### Northern blot analysis

Twenty micrograms of RNA which was extracted with guanidinium thiocyanate were electrophoresed on 1% agarose gel containing formaldehyde and transferred to nylon filters (Sambrook et al, 1989). Blots were hybridized with a $^{32}$P-labelled human S100A4, S100A6 (a gift from Dr DT Denhardt) or glyceraldehyde 3-phosphate dehydrogenase (GAPDH) cDNA which was prepared by the random primer method (Sambrook et al, 1989). Filters were finally washed at 50°C in 0.2 × saline–sodium citrate (SSC) containing 0.1% sodium dodecyl sulphate (SDS).

### Evaluation of S100A4 mRNA stability

Untreated cells and cells treated with 300 IU $ml^{-1}$ IFN-γ for 10 h were incubated with 5 μg $ml^{-1}$ actinomycin D for up to 12 h to block further transcription. Total RNA was extracted and subjected to Northern blot analysis. The blots were hybridized with either an S100A4 cDNA probe or a human GAPDH cDNA probe. After quantifying the intensities of bands by scanning the autoradiograms, the levels of S100A4 mRNA were normalized to those of GAPDH mRNA.

### Nuclear run-off transcription

RNA chains were elongated in nuclei isolated from untreated cells and cells treated with 300 IU $ml^{-1}$ IFN-γ for 20 h. The procedures for isolation of nuclei and nuclear run-off transcription were carried out as described elsewhere (Groudine et al, 1981). Denatured cDNA inserts (4 μg) were immobilized on a single strip of nylon membrane and hybridized to $^{32}$P-labelled RNA transcripts at 42°C for 60 h.

### Fluorescence-activated cell-sorting analysis of IFN-γ receptor (IFN-γ-R) expression

All procedures were carried out at 4°C. Cells were washed three times with Dulbecco's phosphate-buffered saline (PBS) and incubated for 20 min in PBS containing 3% bovine serum albumin (BSA) and 0.1% normal goat serum. The cells were washed with PBS and then incubated for 1 h with either PBS/BSA or a 1:100 dilution of mouse anti-human IFN-γ-R monoclonal antibody, washed, and then incubated for 30 min with a 1:200 dilution of a fluorescein isothiocyanate (FITC)-conjugated goat anti-mouse secondary antibody in PBS/BSA. After washing, cells were fixed with 3% paraformaldehyde and then analysed by fluorescence-activated cell sorting (FACSCAN, Beckton Dickinson, Mountain View, CA, USA).

### Cell motility assay

Cell motility was assessed by phagokinetic track assay as described elsewhere (Takenaga et al, 1994*a*, 1997*a*). Cells were incubated for 24 h in growth medium containing 300 IU $ml^{-1}$ IFN-γ, washed, and then seeded onto coverslips coated with 10 μg $ml^{-1}$ BSA and colloidal gold particle. Cells were seeded at a concentration of 2000 cells per coverslip and incubated for further 24 h in the presence of 300 IU $ml^{-1}$ IFN-γ. The cells were fixed with 3% formaldehyde and then coverslips were mounted. The areas where cells had moved were digitized and measured. Cell motility was evaluated by measuring more than 50 areas free of the gold particles.

### RESULTS

### Effect of IFN-γ on the S100A4 mRNA expression in human colon adenocarcinoma WiDr cells

The effects of IFN-γ and TNF-α on the expression of S100A4 mRNA were examined in WiDr cells, which are reported to express S100A4 (Takenaga et al, 1997*b*). Treatment of the cells with 300 IU $ml^{-1}$ IFN-γ for 48 h resulted in a striking suppression of the S100A4 expression (Figure 1A). However, the expressions of S100A6 and GAPDH mRNAs were not significantly altered by the IFN-γ treatment (Figure 1A). IFN-α (300 IU $ml^{-1}$), IFN-β (300 IU $ml^{-1}$) and TNF-α (10 ng $ml^{-1}$) showed no effect on the S100A4 expression (Figure 1 A,B). These results indicate that the suppression of S100A4 is specific for IFN-γ. The inhibition of the S100A4 expression by IFN-γ was clearly detectable as early as 10 h after the treatment (Figure 2A) and at the concentrations above 200 IU $ml^{-1}$ (Figure 2B). Western blot analysis also revealed a reduction in the S100A4 protein in IFN-γ-treated WiDr cells (Figure 1C). The effects of interleukin 1 (IL-1), IL-6 and various growth factors such as fibroblast growth factor, epidermal growth factor, hepatocyte growth factor and insulin growth factors on the S100A4 expression were also investigated. The results showed that they neither stimulate nor inhibit the S100A4 expression (data not shown).

### Effect of IFN-γ on S100A4 expression and cell surface IFN-γ-R expression in colon adenocarcinoma cells

To examine whether IFN-γ suppresses the expression of S100A4 in other colon adenocarcinoma cells, Colo201 and HT-29 cells, both of which express S100A4 (Takenaga et al, 1997*b*), were incubated with 300 IU $ml^{-1}$ IFN-γ for 48 h. The results in Figure 3 show that IFN-γ significantly suppressed the S100A4 expression in HT-29 cells as observed in WiDr cells, but only weakly in Colo201 cells. The reason might be due to the lower expression of cell surface IFN-γ-R in Colo201 cells than in WiDr and HT-29 cells. To address this question, the cells were incubated with an anti-IFN-γ-R monoclonal antibody followed by incubation with a

© Cancer Research Campaign 1999

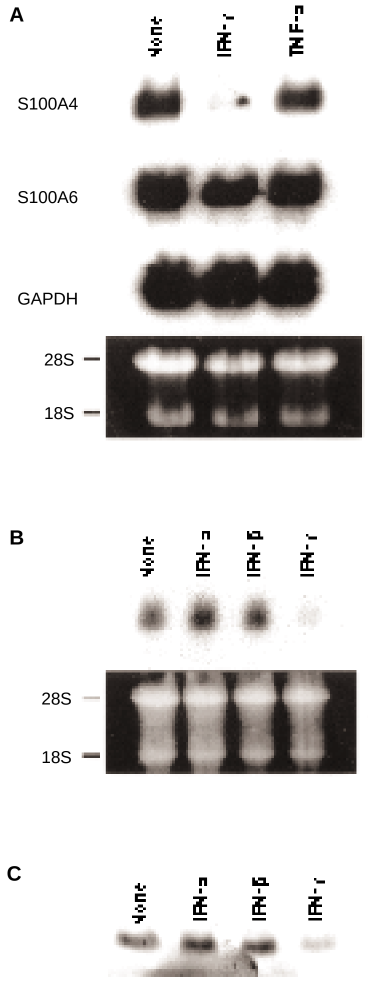

**Figure 1** Effects of interferons on the expression of S100A4 in WiDr cells. (**A**) WiDr cells were cultured in the presence or absence of 300 IU ml$^{-1}$ IFN-γ or 10 ng ml$^{-1}$ TNF-α for 48 h. Total RNA isolated from untreated and cytokine-treated cells was electrophoresed on 1% gel containing formaldehyde and transferred to nylon filters. Blots were hybridized with a $^{32}$P-labelled human S100A4, S100A6 or GAPDH cDNA. Ethidium bromide staining of the gel was also shown. (**B**) WiDr cells were cultured in the presence or absence of 300 IU ml$^{-1}$ IFN-α, IFN-β or IFN-γ for 48 h. RNA blot was hybridized with a $^{32}$P-labelled human S100A4 cDNA. (**C**) WiDr cells were cultured in the presence or absence of 300 IU ml$^{-1}$ IFN-α, IFN-β or IFN-γ for 48 h. Immunoblot analysis was performed as described in Materials and Methods using anti-S100A4 antiserum

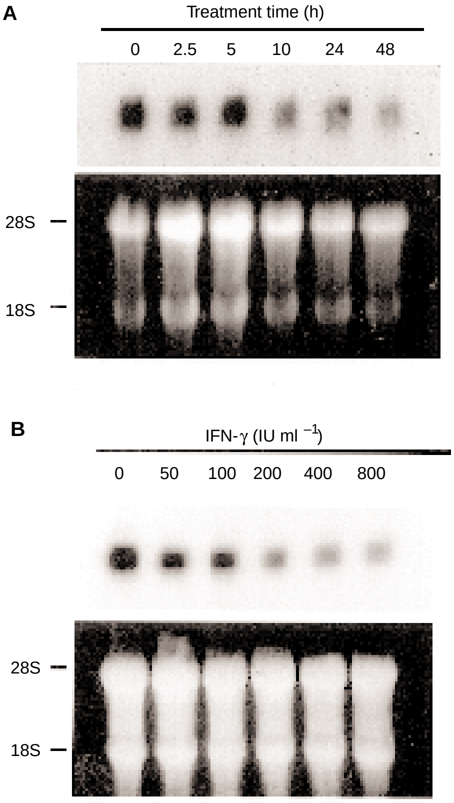

**Figure 2** Effect of IFN-γ on the expression of S100A4 mRNA in WiDr cells. (**A**) Time course. WiDr cells were incubated with 300 IU ml$^{-1}$ IFN-γ for the indicated times. (**B**) Dose–response. WiDr cells were incubated with various concentrations of IFN-γ for 48 h. Total RNA isolated from untreated and IFN-γ-treated cells was subjected to Northern blot analysis. Blots were hybridized with a $^{32}$P-labelled human S100A4 cDNA. Ethidium bromide staining of the gel was also shown fluorescein-conjugated secondary antibody and analysed by fluorescence-activated cell sorting. The results showed that the expression of cell surface IFN-γ-R was indeed lower in Colo201 cells than in WiDr and HT-29 cells (Figure 4).

### Effects of IFN-γ on S100A4 gene transcription and S100A4 mRNA stability in WiDr cells

The molecular mechanism by which IFN-γ down-regulates the steady-state level of S100A4 mRNA was explored by examining the effect of IFN-γ on the S100A4 gene transcription. In vitro transcription analyses showed that the transcriptional rate of S100A4 mRNA as well as that of GAPDH mRNA in IFN-γ-treated cells was indistinguishable from that in untreated cells (Figure 5A). The decay rate of S100A4 mRNA was next compared between WiDr cells and the cells treated with IFN-γ. As shown in Figure 5B, the level of S100A4 mRNA in control cells did not change during a 12-h incubation period with actinomycin D, indicating that the S100A4 mRNA is highly stable as reported previously (Grigorian et al, 1994). On the other hand, the S100A4 mRNA in IFN-γ-treated cells was decayed more rapidly than that in untreated cells. These results suggest that the down-regulation of the steady-state level of the S100A4 mRNA in WiDr cells treated with IFN-γ was partly due to the reduced S100A4 mRNA stability.

### Effect of IFN-γ on cell motility of WiDr cells

WiDr cells were pretreated with 300 IU ml$^{-1}$ IFN-γ for 24 h and then subjected to the motility assay in the presence of IFN-γ for another 24 h. As shown in Figure 6, the motile ability of WiDr cells treated with IFN-γ was significantly lower than that of untreated cells.

### DISCUSSION

The present study has shown that IFN-γ, but not IFN-α and IFN-β, suppressed the expression of S100A4 gene in colon adenocarcinoma cells. Several lines of evidence support that the suppression is not due to its cytotoxicity. First, the expressions of S100A6 and

*© Cancer Research Campaign 1999*

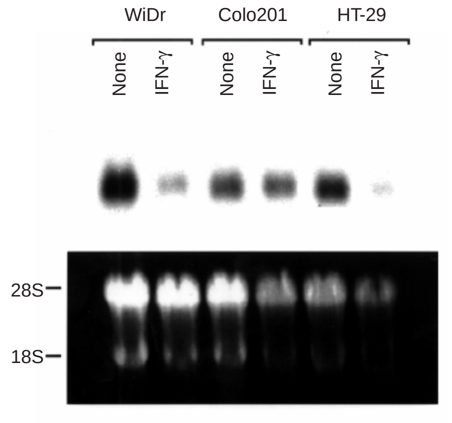

**Figure 3** Effects of IFN-γ on the expression of S100A4 mRNA in various human colorectal carcinoma cells. WiDr, Colo201 and HT-29 cells were incubated with 300 IU ml$^{-1}$ IFN-γ for 48 h. Total RNA isolated from untreated and IFN-γ-treated cells was subjected to Northern blot analysis. Blots were hybridized with a $^{32}$P-labelled human S100A4 cDNA. Ethidium bromide staining of the gel was also shown

GAPDH mRNAs were not significantly suppressed by IFN-γ. Second, TNF-α (10 ng ml$^{-1}$), which inhibited the cell growth to a similar extent as IFN-γ (300 units ml$^{-1}$) (approximately 20%) did not suppress the S100A4 mRNA expression. Third, the suppression of the S100A4 mRNA expression was observed as early as 10h after the IFN-γ treatment, a time when the cells were fully viable.

IFN-γ has been shown to modulate expressions of a variety of genes (Sen and Lengyel, 1992). The mechanisms for the down-regulation of genes caused by IFN-γ are less understood than those for the up-regulation of genes in which the sequences in the 5′-flanking regulatory region, as well as transcription factors, are implicated (Sen et al, 1992). The expression of type II collagen gene has been shown to be down-regulated by IFN-γ, primarily at the transcriptional level (Goldring et al, 1994). On the other hand, genes coding for c-*fos* (Radzich and Varesio, 1991), c-*myc* (Harel-Bellan et al, 1988), transferrin receptor (Bourgeade et al, 1992) and cystic fibrosis transmembrane conductance regulator (Besançon et al, 1994) have been post-transcriptionally modulated by IFN-γ. To examine the mechanisms by which IFN-γ suppresses the S100A4 gene expression, the transcriptional rate of the S100A4 gene and the mRNA stability were examined. The results showed that IFN-γ did not affect its transcription rate but reduced the mRNA stability, indicating that the expression of the S100A4 gene expression in human colon adenocarcinoma cells is down-regulated by IFN-γ at the post-transcriptional level. The mechanism by which IFN-γ reduces the S100A4 mRNA stability is an interesting issue to be solved. Since it has recently been demonstrated that the methylation of S100A4 gene is crucial for the gene expression in human colon adenocarcinoma cell lines (Nakamura and Takenaga, 1998), the DNA methylation status of the S100A4 gene before and after the treatment of WiDr cells with IFN-γ was also examined. However, there was no detectable difference in the degree of DNA methylation between untreated and IFN-γ-treated WiDr cells (data not shown).

Transfection of v-*src*-transformed 3Y1 cells and mouse mammary adenocarcinoma CSMLO cells with S100A4 gene resulted in an increased cell motility (Takenaga et al, 1994*a*; Ford et al, 1995). Conversely, the expression of antisense S100A4 mRNA in S100A4-expressing Lewis lung carcinoma cells lowered cell motility (Takenaga et al, 1997*a*). These results collectively support the assumption that S100A4 participates in regulating cell motility. The present study suggested that IFN-γ-treated WiDr cells became less motile than untreated cells through the suppression of the S100A4 expression. However, the involvement of other factors in the inhibition of cell motility of WiDr cells by IFN-γ cannot be neglected, because IFN-γ possesses a variety of biological effects.

Colo201 cells that express a lower level of IFN-γ-R than WiDr and HT-29 cells were more refractory to IFN-γ in terms of the inhibition of the S100A4 mRNA expression. This result suggests that the inhibition of the S100A4 mRNA expression by IFN-γ is mediated by cell surface IFN-γ-R. If this is the case, an attempt to

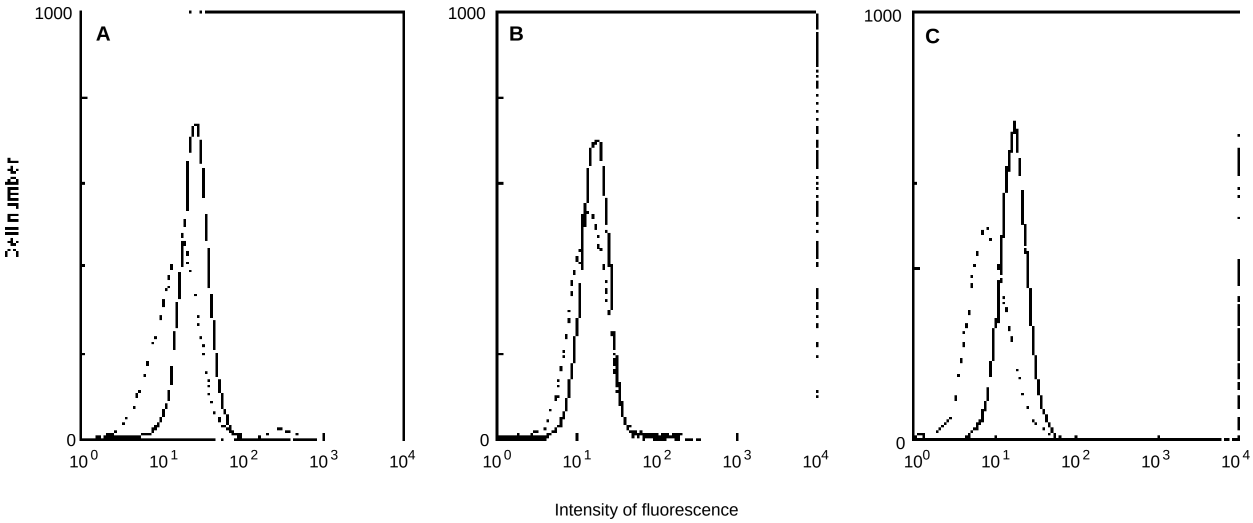

**Figure 4** Flow cytometric analysis of IFN-γ-R expression in various human colorectal carcinoma cells. (**A**) WiDr, (**B**) Colo201, (**C**) HT-29. Cells were incubated in the absence (- - - -) or presence (——) of anti-IFN-γ-R monoclonal antibodies. Cells were then analysed for IFN-γ-R expression as described in Materials and Methods

 *© Cancer Research Campaign 1999*

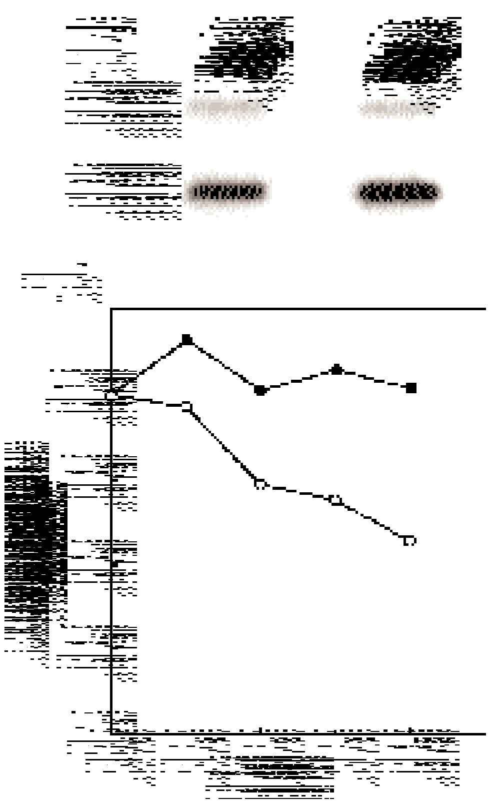

**Figure 5** Effect of IFN-γ on transcription rate of the S100A4 gene and on the stability of S100A4 mRNA in WiDr cells. (**A**) Nuclear run-off analysis of the S100A4 gene transcription in untreated and IFN-γ-treated cells. Nuclei were isolated after 20-h treatment with 300 IU/ml IFN-γ and used for in vitro run-off transcription. Denatured S100A4 or GAPDH cDNA inserts were bound to nylon filters and hybridized to the $^{32}$P-labelled transcripts. (**B**) Stability of S100A4 mRNA in untreated (•) and IFN-γ-treated (○) cells. After 10-h treatment of the cells with 300 IU ml$^{-1}$ IFN-γ, cells were exposed to actinomycin D (5 μg ml$^{-1}$) for the indicated times. RNA blots were hybridized with a $^{32}$P-labelled S100A4 or GAPDH cDNA. The intensities of bands were quantified by scanning the autoradiograms, and S100A4 mRNA levels were normalized to those of GAPDH and expressed as % of control

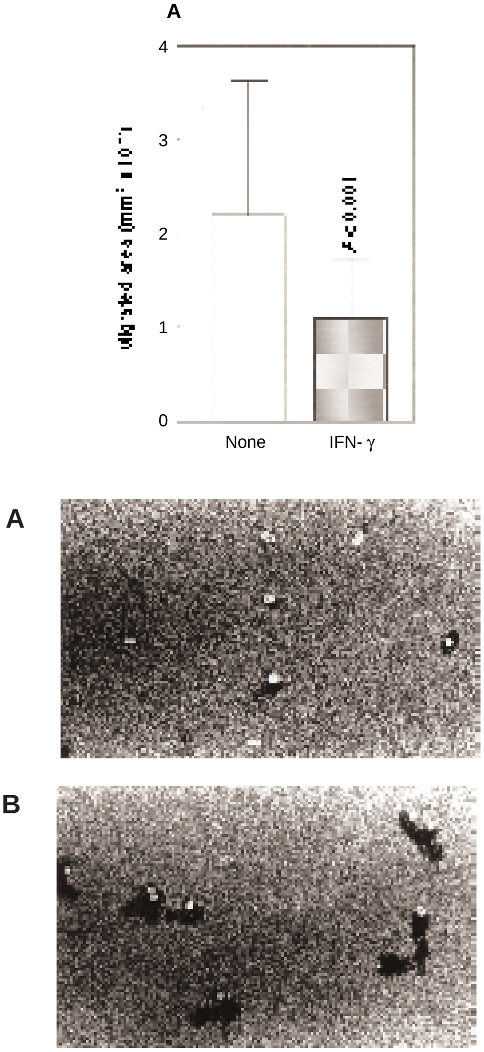

**Figure 6** Effect of IFN-γ on cell motility of WiDr cells. Cells that had been incubated for 24 h in growth medium containing 300 IU ml$^{-1}$ IFN-γ were washed and then subjected to the motility assay for 24 h in the presence of 300 IU ml$^{-1}$ IFN-γ. (**A**) Measured migrated area of untreated and IFN-γ-treated WiDr cells. (**B**) Phagokinetic track patterns produced by untreated (upper panel) and IFN-γ-treated WiDr cells (lower panel)

increase the cell surface IFN-γ-R in Colo201 cells may influence the sensitivity of the cells to IFN-γ. Since it has been reported that IL-1 and TNF-α are able to increase the cell surface IFN-γ-R expression in colon carcinoma cells (Raitano and Korc, 1993), the effects of IL-1 (1000 units per ml) and TNF-α (20 ng ml$^{-1}$) on the cell surface IFN-γ-R expression in Colo201 cells were preliminarily examined. It was found that IL-1 caused an approximately twofold increase in the expression of IFN-γ-R (data not shown). Colo201 cells were then simultaneously treated with IFN-γ and IL-1, and the S100A4 mRNA expression was measured. The results showed that IL-1 augmented the suppressive effect of IFN-γ on S100A4 expression (data not shown). Therefore, the combination treatment of colon adenocarcinoma cells with IFN-γ and IL-1 would be more effective in reducing the S100A4 mRNA expression and thereby in suppressing invasive and metastatic potential of colon adenocarcinoma cells.

Previous reports have shown that IFN-γ shows either a suppressing or an enhancing effect on metastasis of tumour cells. IFN-γ inhibits metastasis of mouse ovarian and lung tumour cells (Ossina et al, 1997), renal adenocarcinoma cells (Yanagihara et al, 1994) and human non-small-cell lung cancer cells (Masumori et al, 1995). By contrast, it enhances experimental metastatic potential of colon 26 adenocarcinoma cells (An et al, 1996) and B16 melanoma cells (Ramani and Balkwill, 1987). These results may be due to the time of treatment of the cells with IFN-γ, as suggested by some investigators (McMillan et al, 1987), or the properties of each cell line. Unfortunately, it is largely unknown whether IFN-γ influences invasiveness and metastasis of human colon adenocarcinoma cells. Therefore, the effects of IFN-γ on these phenotypes should be further clarified.

### ACKNOWLEDGEMENTS

I thank Mr M Hirose for FACScan analysis and Dr S Sakiyama for his critical reading of the manuscript. This work was supported in part by Grants-in-Aid for Cancer Research from the Ministry of Health and Welfare for a New 10-Year Strategy for Cancer Control

*© Cancer Research Campaign 1999* 

of Japan and The Hamaguchi Foundation for the Advancement of Biochemistry.

### REFERENCES

An Z, Wang X, Astoul P, Danays T, Moossa AR and Hoffman RM (1996) Interferon gamma is highly effective against orthotopically-implanted human pleural adenocarcinoma in nude mice. *Anticancer Res* **16**: 2545–2551

Barraclough R, Savin J, Dube SK and Rudland PS (1987) Molecular cloning and sequence of the gene for p9Ka, a cultured myoepithelial cell protein with strong homology to S-100, a calcium-binding protein. *J Mol Biol* **198**: 13–20

Besançon F, Prezewlocki G, Baró I, Hongre A-S, Escande D and Edelman A (1994) Interferon-γ downregulates CFTR gene expression in epithelial cells. *Am J Physiol* **267**: C1398–C1404

Bourgeade M-F, Silbermann F, Kühn U, Testa C, Peschle S, Memet S, Thang M and Besançon F (1992) Post-transcriptional regulation of transferrin receptor mRNA by IFN-γ. *Nucleic Acids Res* **20**: 2997–3003

Celio MR, Pauls T and Schwaller B (1996) *Guidebook to the Calcium-Binding Proteins*, Sambrook and Tooze (eds). Oxford University Press: Oxford

Davies BR, Davies MPA, Gibbs FEM, Barraclough R and Rudland PS (1993) Induction of the metastatic phenotype by transfection of a benign rat mammary epithelial cell line with the gene for p9Ka, a rat calcium-binding protein, but not with the oncogene EJ-*ras*-1. *Oncogene* **8**: 999–1008

Ebralidze A, Tulchinsky E, Grigorian M, Afanasyeva A, Senin V, Revazova E and Lukanidin E (1989) Isolation and characterization of a gene specifically expressed in different metastatic cells and whose deduced gene product has a high degree of homology to a $Ca^{2+}$-binding protein family. *Genes Dev* **3**: 1086–1093

Ford HL and Zain SB (1995) Interaction of metastasis-associated mts 1 protein with non-muscle myosin. *Oncogene* **10**: 1597–1605

Ford HL, Salim MM, Chakravarty R, Aluiddin V and Zain SB (1995) Expression of mts1, a metastasis-associated gene, increases motility but not invasion of a nonmetastatic mouse mammary adenocarcinoma cell line. *Oncogene* **11**: 2067–2075

Goldring MB, Fukuo K, Birkhead JR, Dudek E and Sandell LJ (1994) Transcriptional suppression by interleukin-1 and interferon-γ of type II collagen gene expression in human chondrocytes. *J Cell Biochem* **54**: 85–99

Goto K, Endo H and Fujiyoshi T (1988) Cloning of the sequences expressed abundantly in established cell lines: identification of a cDNA clone highly homologous to S-100, a calcium binding protein. *J Biochem* **103**: 48–53

Grigorian MS, Tulchinsky EM, Zain S, Ebralidze AK, Kramerov DA, Kriajevska MV, Georgiev GP and Lukanidin EM (1993) The mts1 gene and control of tumor metastasis. *Gene* **135**: 229–238

Grigorian M, Tulchinsky E, Burrone O, Tarabykina S, Georgiev G and Lukanidine E (1994) Modulation of mts 1 expression in mouse and human normal and tumour cells. *Electrophoresis* **15**: 463–468

Grigorian M, Ambartsumian N, Lykkesfeldt AE, Bastholm L, Elling F, Georgiev G and Lukanidin E (1996) Effect of mts1 (S100A4) expression on the progression of human breast cancer cells. *Int J Cancer* **67**: 831–841

Groudine M, Peretz M and Weintraub H (1981) Transcriptional regulation of hemoglobin switching on chicken embryos. *Mol Cell Biol* **1**: 281–288

Harel-Bellan A, Brini AT and Farrar WL (1988) Interferon-gamma inhibits c-*myc* gene expression by impairing the splice process in a colony-stimulating factors dependent murine myeloid cell line. *J Immunol* **141**: 1012–1017

Hilt DC and Kligman D (1991) The S-100 protein family: a biochemical and functional overview. In *Novel Calcium-binding proteins*. *Fundamental and Clinical Implications*, Heizman CW (ed), pp. 65–103. Springer-Verlag: Berlin

Ilg EC, Schäfer BW and Heizmann CW (1996) Expression pattern of S100 calcium-binding proteins in human tumors. *Int J Cancer* **68**: 325–332

Jackson-Grusby LL, Swiergiel J and Linzer DIH (1987) A growth-related mRNA in cultured mouse cells encodes a placental calcium-binding protein. *Nucleic Acids Res* **15**: 6677–6690

Kriajevska MV, Cardenas MN, Grigorian MS, Ambartsumian NS, Georgiev GP and Lukanidin EM (1994) Non-muscle myosin heavy chain as a possible target for protein encoded by metastasis-related mts-1 gene. *J Biol Chem* **269**: 19679–19682

McMillan TJ, Rao J, Everett CA and Hart IR (1987) Interferon-induced alterations in metastatic capacity, class-1 antigen expression and natural killer cell sensitivity of melanoma cells. *Int J Cancer* **40**: 659–663

Masumori N, Tsukamoto T and Kumamoto Y (1995) Interferon-gamma and interleukin-2 suppress the experimental metastasis of mouse renal adenocarcinoma. *Int J Urol* **2**: 6–11

Maelandsmo GM, Hovig E, Skrede M, Engebraaten O, Flørens VA, Myklebost O, Grigorian M, Lukanidin E, Scanlon KJ and Fodstad Ø (1996) Reversal of the in vivo metastatic phenotype of human tumor cells by anti-CAPL (mts1) ribozyme. *Cancer Res* **56**: 5490–5498

Nakamura N and Takenaga K (1998) Hypomethylation of the metastasis-associated S100A4 gene correlates with gene activation in human colon adenocarcinoma cell lines. *Clin Exp Metastasis* **16**: 471–479

Ossina NK, Cannas A, Powers VC, Fitzpatrick PA, Knight JD, Gilbert JR, Shekhtman EM, Tomei LD, Umansky SR and Kiefer MC (1997) Interferon-gamma modulates a p53-independent apoptotic pathway and apoptosis-related gene expression. *J Biol Chem* **272**: 16351–16357

Parker C, Whittaker PA, Usmani BA, Lakshmi MS and Sherbet GV (1994) Induction of 18A2/mts 1 gene expression and its effect on metastasis and cell cycle control. *DNA Cell Biol* **13**: 1021–1028

Pedrocchi M, Schäfer BW, Mueller H, Eppenberger U and Heinzmann CW (1994) Expression of $Ca^{2+}$-binding proteins of the S100 family in malignant human breast-cancer cell lines and biopsy samples. *Int J Cancer* **57**: 684–690

Radzich D and Varesio L (1991) c-fos mRNA expression in macrophages is down-regulated by interferon-γ at the post-transcriptional level. *Mol Cell Biol* **11**: 2718–2722

Radinsky R (1995) Modulation of tumor cell gene expression and phenotype by the organ-specific metastatic environment. *Cancer Metastasis Rev* **14**: 323–338

Raitano AB and Korc M (1993) Growth inhibition of a human colorectal carcinoma cell line by interleukin 1 is associated with enhanced expression of γ-interferon receptors. *Cancer Res* **53**: 636–640

Ramani P and Balkwill FR (1987) Enhanced metastasis of a mouse carcinoma after in vitro treatment with murine interferon gamma. *Int J Cancer* **40**: 830–834

Sambrook J, Fritsch EF and Maniatis T (1989) *Molecular Cloning*: *A Laboratory Manual*, 2nd Edn. Cold Spring Harbor Laboratory: New York

Schäfer BW and Heizmann CW (1996) The S100 family of EF-hand calcium-binding proteins: functions and pathology. *Trends Biochem Sci* **21**: 134–140

Sen GC and Lengyel P (1992) The interferon system. *J Biol Chem* **267**: 5017–5020

Strutz F, Okada H, Lo CW, Danoff T, Carone RL, Tomaszewski JE and Neilson EG (1995) Identification and characterization of a fibroblast marker: FSP1. *J Cell Biol* **130**: 393–405

Takenaga K, Nakamura Y, Endo H and Sakiyama S (1994*a*) Involvement of S100-related calcium-binding protein pEL98 (or mts 1) in cell motility and tumor cell invasion. *Jpn J Cancer Res* **85**: 831–839

Takenaga K, Nakamura Y and Sakiyama S (1994*b*) Expression of a calcium binding protein pEL98 (mts1) during differentiation of human promyelocytic leukemia HL-60 cells. *Biochem Biophys Res Commun* **202**: 94–101

Takenaga K, Nakamura Y and Sakiyama S (1994*c*) Cellular localization of pEL98 protein, an S100-related calcium binding protein, in fibroblasts and its tissue distribution analyzed by monoclonal antibodies. *Cell Struct Funct* **19**: 133–141

Takenaga K, Nakamura Y, Sakiyama S, Hasegawa Y, Sato K and Endo H (1994*d*) Binding of pEL98 protein, an S100-related calcium-binding protein, to nonmuscle tropomyosin. *J Cell Biol* **124**: 757–768

Takenaga K, Nakamura Y and Sakiyama S (1997*a*) Expression of antisense RNA to S100A4 gene encoding an S100-related calcium-binding protein suppresses metastatic potential of high-metastatic Lewis lung carcinoma cells. *Oncogene* **14**: 331–337

Takenaga K, Nakanishi H, Wada K, Suzuki M, Matsuzaki O, Matuura A and Endo H (1997*b*) Increased expression of S100A4, a metastasis-associated gene, in human colorectal adenocarcinomas. *Clin Cancer Res* **3**: 2309–2316

Yanagihara K, Seyama T and Watanabe Y (1994) Antitumor potential of interferon-γ retroviral expression of mouse interferon-γ cDNA in two kinds of highly metastatic mouse tumor lines reduces their tumorigenicity. *Nat Immunol* **13** 102–112

 *© Cancer Research Campaign 1999*
